# Ventricular Septal Dissection Complicating Inferior Wall Myocardial Infarction

**DOI:** 10.1155/2017/9680891

**Published:** 2017-03-15

**Authors:** Lindsey Kalvin, Rayan Yousefzai, Bijoy K. Khandheria, Timothy E. Paterick, Khawaja Afzal Ammar

**Affiliations:** Aurora Cardiovascular Services, Aurora Sinai/Aurora St. Luke's Medical Centers, 2801 W. Kinnickinnic River Parkway, Milwaukee, WI, USA

## Abstract

Postmyocardial infarction ventricular septal defect is an increasingly rare mechanical complication of acute myocardial infarction. We present a case of acute myocardial infarction from right coronary artery occlusion that developed hypotension and systolic murmur 12 hours after successful percutaneous coronary intervention. Although preoperative imaging suggested a large ventricular septal defect and a pseudoaneurysm, intraoperative findings concluded a serpiginous dissection of the ventricular septum. The imaging technicalities are discussed.

## 1. Introduction

Postmyocardial infarction ventricular septal defect (VSD) is a rare complication (1/1000), and ventricular septal dissection is an even less common complication with only four case reports previously described [1–4], the first occurring in 1988. None of these cases appeared on imaging like a cross between a pseudoaneurysm and a VSD, a finding unique to our case.

## 2. Case Presentation

**Figure 1:**
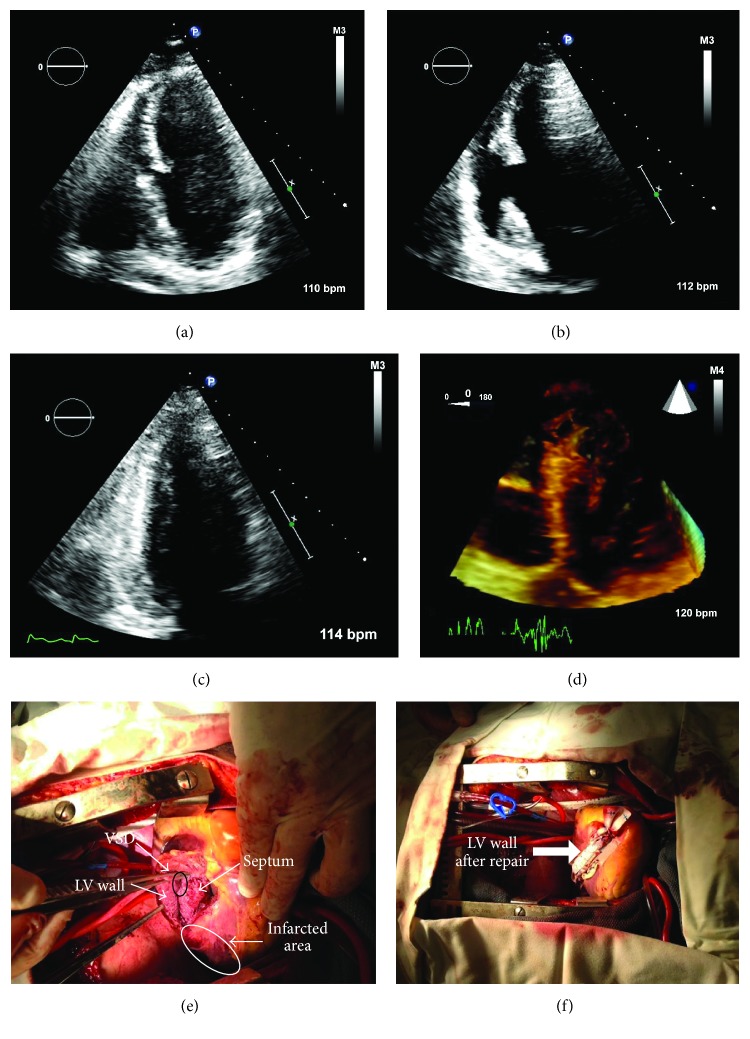
Four-chamber echocardiographic views of an apparent ventricular septal defect of the inferior septum (a) and an apparent pseudoaneurysm of the inferior wall (b), which persisted despite rotating the transducer 90 degrees or more from the four-chamber apical window. On repeat imaging, a very narrow apical window was identified in which the two-chamber view was seen without the pseudoaneurysm (c). Also shown is a three-dimensional echocardiographic depiction of the serpiginous nature of the ventricular septal dissection (d). Surgical view of the infarcted area and partial ventricular septal defect (e). Surgical view of the septum covered by a Dacron patch (f).

A 60-year-old man presented with chest pain and inferior-lead ST-segment elevation on electrocardiography. He was found to have right coronary artery occlusion and underwent successful percutaneous intervention with a drug-eluting stent. Twelve hours later, he developed mild hypotension (80/60 mmHg) and a II/VI holosystolic murmur. Transthoracic echocardiography revealed a large ventricular septal defect (VSD) in the mid-inferior septal wall (Figure 1(a), Video 1 in Supplementary Material available online at https://doi.org/10.1155/2017/9680891) as well as a pseudoaneurysm in the mid-inferior wall, which persisted despite rotating the transducer 90 degrees from the apical four-chamber view (Figure 1(b), Video 2). On repeat imaging, a very narrow apical two-chamber view window in which the inferior wall was intact without the pseudoaneurysm (Figure 1(c), Video 3) was identified with difficulty. Immediate surgery did not reveal a pseudoaneurysm of the inferior wall despite detailed surgical interrogation. The location of the apparent VSD was covered by an aneurysmal structure, with no communication between the right and left ventricles. It was felt that the patient had a serpiginous dissection of the infarcted inferior septum (Figure 1(d), Video 4), which took on the appearance of a pseudoaneurysm on echocardiography. The partial defect in the septum was surgically covered by a Dacron patch (Figures 1(e) and 1(f)) during surgery, which was performed within 24 hours of the diagnostic echocardiogram. The operation resolved the imaging abnormalities seen preoperatively.

## 3. Discussion

The prior case reports included use of three-dimensional transesophageal echocardiography, which ably depicted the serpiginous tract in our patient. Perhaps the harsh systolic murmur typically seen in postinfarction VSD was only of lower grade here because it was generated from flow across the left ventricle to the intramural interventricular septum cavity/dissection without communication with the right ventricle. We hypothesized that the ventricular septum had septal perforators from both the right coronary artery (infarct-related artery) and left anterior descending artery, with no flow for more than 24 hours in the septal perforators from the right coronary artery and normal blood flow in perforators from the left anterior descending artery. This created shearing forces at the “junction” of the hyperkinetic septum (perfused from the left anterior descending artery) and the akinetic septum (perfused from the right coronary artery). The shearing forces caused dissection of the septum along the junction lines, creating a partial VSD. The right ventricular side of the septum stayed intact and formed an aneurysmal sac. The large pseudoaneurysm-like structure was indeed an intramural hematoma/dissection of the interventricular septum open only on the left ventricular side. Although this mechanism was not described in the prior case reports on ventricular septal dissection [1–4], pseudoaneurysm formation and free-wall rupture in the ventricular free wall are known to occur as a result of this mechanism. In a recent case report, this was noted in a patient who underwent postmyocardial infarction VSD repair and then developed septal dissection as the necrotic tissue in the septum failed to heal [5], necessitating repeat surgical repair. Although echocardiography was useful, MRI provided incremental insights into this case. Rarely, septal dissection can be due to rupture of other surrounding structures, such as ruptured sinus of Valsalva extending into the septum [6].

Both postmyocardial infarction entities, pseudoaneurysm and VSD, can be lethal, with mortality rates of approximately 90% unless immediate diagnosis leads to surgical correction. Since most cases of ventricular septum dissection in the literature are with inferior infarction, clinicians need to stay alert for this complication in cases of inferior infarction. Both the left anterior descending and right coronary arteries have septal perforators, anterior septal versus inferior septal, respectively. Therefore occlusion of either can potentially lead to this complication.

## Supplementary Material

Video 1 shows a four-chamber echocardiographic view of an apparent ventricular septal defect of the inferior septum. Video 2 is of a two-chamber echocardiographic view of an apparent pseudoaneurysm of the inferior wall, which persisted despite rotating the transducer 90 degrees. Video 3 shows a narrow two-chamber echocardiographic view without the pseudoaneurysm. Video 4 is a three-dimensional depiction of the serpiginous nature of the patient's ventricular septal dissection.
