# Supplementary material for: Ventricular Septal Dissection Complicating Inferior Wall Myocardial Infarction
Source: Case Rep Cardiol. 2017 Mar 15;2017:9680891. doi: 10.1155/2017/9680891 (PMC5371196; doi:10.1155/2017/9680891)
Supplement: Supplementary file 1 — Video 1 shows a four-chamber echocardiographic view of an apparent ventricular septal defect of the inferior septum. Video 2 is of a two-chamber echocardiographic view of an apparent pseudoaneurysm of the inferior wall, which persisted despite rotating the transducer 90 degrees. Video 3 shows a narrow two-chamber echocardiographic view without the pseudoaneurysm. Video 4 is a three-dimensional depiction of the serpiginous nature of the patient's ventricular septal dissection. [file 9680891.f1.zip › mat.9680891.v1.doc]

**Supplemental Material**

**Movie Legends**

**Video 1.** Four-chamber echocardiographic view of an apparent ventricular septal defect of the inferior septum.

**Video 2.** Two-chamber echocardiographic view of an apparent pseudoaneurysm of the inferior wall, which persisted despite rotating the transducer 90 degrees.

**Video 3.** Narrow two-chamber echocardiographic view without the pseudoaneurysm.

**Video 4.** Three-dimensional depiction of the serpiginous nature of the patient’s ventricular septal dissection.
